# Novel properties of phenanthroline‐amine include binding to amyloid plaques and suppressing plaque growth in APP/PS1 mouse model of Alzheimer’s disease (AD)

**DOI:** 10.1002/alz70859_103900

**Published:** 2025-12-25

**Authors:** Debomoy K. Lahiri, Bryan Maloney, Calvert Schmued, Larry Schmued

**Affiliations:** ^1^ Indiana Alzheimer's Disease Research Center, Indianapolis, IN USA; ^2^ Indiana University School of Medicine, Department of Psychiatry, Indianapolis, IN USA; ^3^ Histo‐Chem Inc, White Hall, AR USA

## Abstract

**Background:**

Amyloid‐β precursor protein (APP) and apolipoprotein E (APOE) are both involved in AD. APP is proteolytically cleaved to produce neurotoxic amyloid fragments. Screening a variety of compounds for their ability to reduce the amyloid plaque burden in mice models of AD provides potential novel drug targets. One promising class of compounds are certain metal chelators including phenanthroline‐amine (PAA), a metal chelator, an aromatic cation and a metalloprotease inhibitor shown to significantly reduce the size and number of plaques seen in year old APP/PS1 mice (Schmued et al‐2024). Our objective was to expand the previous report of APP administration using a double transgenic model (N=16) that express considerably more plaques. The second objective of this study was to determine whether PAA will bind directly to amyloid plaques in tissue sections.

**Method:**

At 1 year of age both PAA‐dosed and control APP/PS1 mice were euthanized, and their fixed brains removed and freeze‐sectioned. Some sections were stained with hydroxyquinoline oxalate to label all amyloid plaques. In parallel labeling studies, the tissue sections were immersed in PAA dissolved in a pH 4.5 buffer at 55^o^C. Double labeling was also achieved by combining these two staining methods on the same tissue section.

**Result:**

Daily oral dosing with PAA resulted in a significant reduction in the number and size of amyloid plaques vs. the vehicle treated control mice. The average plaque areas of the PAA treated group exhibited 67.6% of the plaque burden seen in the control animals. Also, immersing tissue sections in a PAA solution resulted in the red fluorescent labeling of all amyloid plaques. Double labeling revealed that PAA labeled the plaques more extensively than HQ‐O.

**Conclusion:**

Our study confirmed that chronic oral dosing with PAA resulted in plaque reductions comparable to that seen in the original study that used a smaller number of a less common mouse model of AD. This study also showed that PAA is capable of binding directly to amyloid plaques. Possible endogenous target molecules, based on the chemical properties of PAA, include certain transition metals, certain glycosylated molecules of AD relevance (APOE, NCAM1, APP and gangliosides) and metalloproteases.

